# The physiological, musculoskeletal and psychological effects of stand up paddle boarding

**DOI:** 10.1186/s13102-016-0057-6

**Published:** 2016-10-10

**Authors:** Ben Schram, Wayne Hing, Mike Climstein

**Affiliations:** 1 School of Physiotherapy, Faculty of Health Science & Medicine, Bond University, Gold Coast, QLD 4229 Australia; 2 Water Based Research Unit, Bond Institute of Health and Sport, Faculty of Health Science & Medicine, Bond University, Gold Coast, QLD 4229 Australia; 3 Exercise, Health & Performance Faculty Research Group, Faculty of Health Sciences, the University of Sydney, Sydney, NSW Australia

**Keywords:** Training intervention, Water sports, Aquatic, Paddle boarding, SUP

## Abstract

**Background:**

Stand up paddle boarding (SUP) is a rapidly growing sport and recreational activity where anecdotal evidence exists for its proposed health, fitness and injury rehabilitation benefits. While limited scientific evidence exists to substantiate these claims, previous studies have shown that high levels of fitness, strength and balance exists amongst participants of this sport. The purpose of this study was to conduct a training intervention on a group of previously untrained individuals to ascertain the potential of SUP on various health parameters.

**Methods:**

An intervention study was conducted where after being tested initially, subjects were left for 6 weeks to act as their own control before the SUP intervention began. A total of 13 SUP participants completed the training study (nine males, four females) which was comprised of three 1 h sessions per week for 6 weeks.

**Results:**

No significant changes occurred during the initial control period. Significant (*P* < 0.05) improvements were made in aerobic (+23.57 %) and anaerobic fitness (+41.98 %), multidirectional core strength tests (prone +19.78 %, right side +26.19 %, left side +28.31 %, Biering Sorensen +21.33 %) and self-rated quality of life questionnaires in the physical (+19.99 %) and psychological (+17.49 %) domains. No significant changes were detected in static or dynamic balance over the duration of the training intervention.

**Conclusion:**

These results demonstrate the cardiovascular, musculoskeletal and psychological improvements achievable for the novice when utilizing SUP as a training tool. The result from this study provides some evidence to substantiate the claims of health and fitness benefits SUP.

## Background

Stand up paddle boarding (SUP) originated in Hawaii in the 50's and is a mixture of both surfing and paddling [1]. It is an emerging recreational activity which has attracted attention for its proposed fitness, strength and balance benefits. Anecdotally, SUP is thought to be a beneficial clinical training tool as it possesses many facets of an ideal rehabilitative exercise [2]. However our recent review of the literature has identified minimal scientific evidence to substantiate the proposed benefits.

Stand up paddle boarding is a physical activity in which the participant maintains a standing position on a board similar to a surfboard. However, stand up paddle boards are longer in length (8–15', 2.44–4.57 m), thicker (4–8", 10.16–20.32 cm) and wider (26–31", 66.04–78.74 cm) than traditional surfboards. Stand up paddle boarding involves a participant getting to their feet on a large board before using the long paddle for propulsion with strokes on either side of the body [3]. Paddling involves the similar biomechanics of dragon boat racing which has the paddling mechanics of an entry, drive and exit of the paddle from the water [4]. It requires a rhythmic alternating paddle to propel the craft through the water. Isometric contractions of the entire trunk, gluteals and lower leg musculature are required to counter the rotational forces from the pull phase of each paddling stroke [2].

One of the major attractions of SUP is that it is thought to a good fitness training tool. Physical activity is well understood to increase cardiovascular fitness which is associated with cardiovascular mortality [5]. Physical inactivity is a major modifiable risk factor of a range of non‐communicable diseases such as diabetes mellitus, osteoporosis and some forms of cancer [6]. Physical activity significantly improves overall health, lowers the risk of heart disease by 40 %, stroke by 27 % and lowers the incidence of hypertension by almost 50 % [7]. Physical activity has also been associated with improved mental health and well‐being, minimizing the risk of developing Alzheimer’s and depression [6].

Our prior research has demonstrated that high levels of aerobic and anaerobic fitness, core strength and balance are possessed by those classed as elite amongst this sport [8, 9]. Given the issue of sedentary behaviour and limited scientific research on SUP regarding the anecdotal claims of benefit of this activity, our intention was to assess the benefit of SUP on a group of sedentary, untrained individuals with respect to fitness, strength, balance and self-rated quality of life.

## Methods

**Fig. 1:**
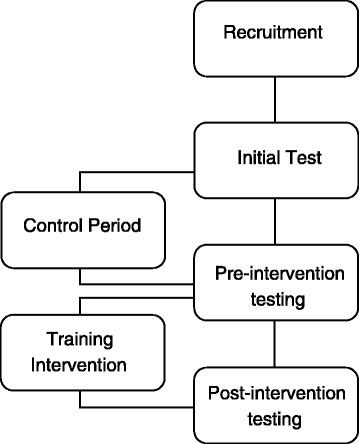
Study design

A training study in which the aerobic and anaerobic capacity, blood lipid profile, body composition, isometric core strength, static and dynamic balance ability of a group of sedentary individuals was monitored pre and post an intervention period of 6 weeks (Fig. 1). These measures were recorded initially, before a 6-week control period, before the training intervention and post intervention to determine the effect of SUP on the various health and well-being parameters.

A total of 18 sedentary individuals (ten males, eight females) were recruited through radio and media advertisements about the study. A total of 13 individuals (four females, nine males) completed the training program. Inclusion criteria required individuals to have not been participating in physical activity for the last 6 months and were aged between 18 and 60 years. Exclusion criteria included a history of back pain, physical and psychological impairment. The study was approved by the University Human Research Ethics Committee (RO-1550) and each participant formally consented to taking part in the study.

The training program consisted of 3 1 h sessions per week for 6 weeks. There was a rest period of 48 h before subsequent sessions with no sessions being performed on Sundays for either group. Participants were given longer, wider boards to begin with (~11' length, 33" width, 4.6" thickness), before moving on to shorter, narrower boards (~length 9'1, 29.5"width, 4.4" thickness) to challenge postural control more as the weeks progressed. The intensity of the sessions was gradually increased until week three where high intensity sprint based training was incorporated into the week with the long slow sessions. Initially, participants were paddling 1 km in an endurance session, which increased to 10 km by then end of the training program. High intensity initially involved 2 min of 10 s paddling, 10 s resting which progressed to 5 min of 10 s on, 10 s off. Given the similar, low initial fitness of the males and females, the same training program was provided to all participants of this study. The majority of the training sessions were performed on the SUP boards with some minimal land based training which included running from the shore to the SUP boards to begin a paddle session. Participants were instructed to perform no other physical activity apart from the SUP training during the interventional period.

For testing, participants attended the human performance laboratory where they were assessed for height and weight on a standard medical balance scale (Seca, 700, Hamburg, Deutschland). Body composition and basal metabolic rate was assessed using bio-electrical impedance (Tanita Body Composition Analyzer MC-980MA, Illinois, USA) as this has been shown to successfully determine body composition [10]. Bloods lipids were analysed prior to exercise using a portable analyser (Cardiochek, P.A. Indiana, USA) to ascertain total cholesterol (TC), high density lipoproteins (HDL), low density lipoproteins (LDL) and triglycerides (Trigs). The Cardiochek is Cholesterol Reference Method Laboratory Network (CRMLN) certified and has high correlation to standard venous blood samples [11].

**Fig. 2:**
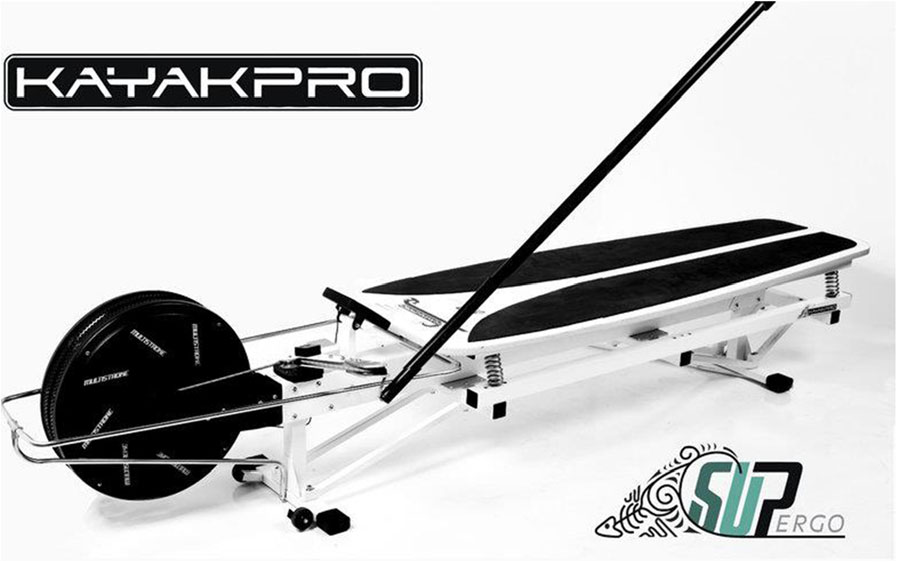
The KayakPro SUPErgometer

A continuous graded exercise test on a specialised SUP ergometer (KayakPro SUPErgo, Miami, FL, USA) was used to determine maximal aerobic power (relative and absolute). The SUPErgo is elevated on springs which aims to replicate the instability of paddling on water (Fig. 2). This laboratory assessment has previously been shown to correlate highly to field based measures [12]. Maximal aerobic power (VO_2max_) was determined using an automated expired gas analysis system (Parvomedics TrueOne 2400 metabolic system, East Sandy, Utah, USA) which was calibrated prior to each test. The expired-gas-analysis system meets Australian Institute of Sport accreditation standards for precision and accuracy. The gas analysis software was configured to breath by breath however VO_2_ max was determined from the average of 30 s of max data collected.

The SUP ergometer VO_2_max protocol involved participants starting at an initial power output of 5 W with a 2 W increase each minute until volitional exhaustion. Participants were instructed to paddle as per normal, free to alternate paddling on each side ad libitum.

On the subsequent visit to the laboratory, maximal anaerobic power was determined using the same SUP ergometer (KayakPro SUPErgo, USA). Participants were allowed to choose their preferred paddling side on the ergometer to ensure that an indication of their maximal power output could be reached. Participants then paddled maximally for 10 s from a stationary start. The maximal power was then determined using specialised software incorporated into the SUP ergometer (eMonitor Pro 2 KayakPro, New Rochelle, NY, USA) which is interfaced with a computer. Other anaerobic power parameters measured included distance covered in 10 s and peak anaerobic speed. Participant heart rates were monitored with a 12 lead ECG via telemetry during both maximal tests. A minimum of 2 days and a maximum of 3 days were allowed between testing days.

Postural control was assessed in the laboratory via a portable force platform (Kistler 2812D with Bioware 4.0, 100 Hz sampling rate) with three piezoelectric force sensors used to calculate the centre of pressure (COP) foot positions. Measurements using such force platforms have been shown to be reliable, giving data such as length, area and velocity of sway [13]. Static and dynamic postural control was assessed as per Palliard [14]. Static postural control was assessed for 50 s while dynamic postural control on a seesaw was assessed for 25 s. These conditions were tested with eyes open (EO) and then repeated with eyes closed (EC). The tests were conducted in order from most stable to least stable.

COP signals were smoothed using a Butterworth filter with a 10Hz low pass cut off frequency. The 100 % square (a square in which all the samples lie) was calculated post collection via the range of both the x and y deviations. The COP sway path length (the total distance travelled by the COP over the course of the trial duration) was calculated via the distance between each sampling point. From the COP excursion, the COP velocity was calculated (Velocity = Distance/Time).

Trunk muscle endurance assessments were performed as per McGill in which the flexors of the spine were assessed with a prone bridge, the lateral flexors with the side bridge and the extensors with the Biering Sorensen test [15]. The tests were terminated when the participant could no longer hold the horizontal position as determined by the tester and the time was recorded.

Finally a self-rated quality of life questionnaire (WHO-QoL Bref UK edition) was completed by the participants pre and post training program [16]. It comprises of 26 items across 4 domains of physical health psychological health, social relationships and environment. It was administered pre and post intervention to assess the effect of a SUP intervention on self-rated quality of life measures.

### Data analysis

All statistical analyses were completed using the IBM Statistical Package for the Social Sciences (SPSS, Version 20.0) software program (mean ± SD) comparing initial test, pre and post testing for the groups. Normality was assessed via Shapiro-Wilk (*P* < 0.05) and visual inspection of the data’s histogram and tests of sphericity were also performed to ensure minimising type 1 errors. Peak Speed, stroke rate, anaerobic peak speed and anaerobic distance covered were deemed to not be a normally distributed, therefore a Friedman Test was utilised with a post hoc Wilcoxon Signed Ranks test was utilised. All other data was deemed to be normally distributed and therefore a repeated measure ANOVA was utilised with a Bonferonni adjustment for multiple comparisons performed post hoc to determine statistical significance between groups.

## Results

Thirteen participants (46.15 ± 11.63 years, 173.79 ± 10.53 cm) completed the training intervention. An overall attendance rate of 90.27 % was seen throughout the training program with the primary reasons for missing sessions being family commitments, sickness and minor injury. Of the five participants who did not complete the training program, one suffered an injury which resulted in them withdrawing from the study, another was unable to attend training sessions due to changes in work schedule, one participant from sickness and the other two did not provide reasons. The injured paddler suffered an aggravation of a pre-existing rib injury which was not associated with SUP.

There were no significant differences between initial and subsequent testing prior to beginning the training intervention. Participants were classified as being overweight according to their Body Mass Index (BMI) throughout the training period (Table 1).

**Table 1:** Body composition and blood profiling

	Initial test	Pre-training	Post-training
*Body Composition*			
Weight (kg)	84.76 ± 17.22	85.45 ± 17.96	84.91 ± 16.51
BMI (kg/m^2^)	27.98 ± 4.72	28.17 ± 4.82	28.02 ± 4.38
Body fat (%)	26.33 ± 5.15	26.74 ± 5.47	26.41 ± 5.13
*Blood Profiling*			
Total cholesterol (mmol/L)	4.67 ± 0.65	4.72 ± 0.73	4.89 ± 0.68
HDL (mmol/L)	1.34 ± 0.48	1.39 ± 0.61	1.61 ± 0.52
Triglycerides (mmol/L)	1.46 ± 0.51	1.44 ± 0.93	1.54 ± 0.79
LDL (mmol/L)	2.71 ± 0.67	2.77 ± 0.51	2.58 ± 0.84

Mean ± SD

Table 2 shows many improvements with respect to aerobic fitness. There were significant improvements in absolute aerobic power (+18.86 %) and relative aerobic power (+23.57 %) at the completion of the training *(p* < 0.05*)*. There were no significant changes in RER, HRmax, and average or peak stroke length. Increases in distance covered (+44.79 %) and peak speed (+10.26 %) were observed. There was a weak, negative correlation between age of the participants and % of VO_2_ max increase over the study (*r* = −0.32).

**Table 2:** Physiological results

	Initial test	Pre-training	Post training
*Aerobic Performance*			
VO_2max_ (L/min)	1.79 ± 0.59	1.75 ± 0.56	2.08 ± 0.56*
VO_2max_ (ml/kg/min)	20.25 ± 3.92	19.68 ± 3.70	24.32 ± 4.22*
Respiratory exchange ratio	1.16 ± 0.07	1.18 ± 0.07	1.14 ± 0.07
HR_peak_ (bpm)	171.46 ± 16.72	172.31 ± 16.10	171.23 ± 15.14
Aerobic power (W)	10.52 ± 3.05	11.51 ± 3.24	15.20 ± 3.13*
Average Stroke Length (m)	2.38 ± 0.46	2.42 ± 0.48	2.52 ± 0.40
Peak Stroke Length (m)	2.89 ± 0.66	2.93 ± 0.71	2.96 ± 0.61
Peak stroke rate (strokes/min)	41.15 ± 9.10	39.62 ± 5.20	43.77 ± 4.71
Distance covered (m)	366.68 ± 71.90	336.21 ± 101.97	486.80 ± 134.64*
Peak speed (m/s)	1.51 ± 0.15	1.56 ± 0.14	1.72 ± 0.12*
*Anaerobic Performance*			
Absolute power output (W)	14.08 ± 6.68	16.58 ± 7.72	23.54 ± 7.91*
Relative power output (W/kg)	0.16 ± 0.06	0.19 ± 0.07	0.27 ± 0.07*
Peak speed (m/s)	1.60 ± 0.32	1.71 ± 0.32	1.98 ± 0.22*
Distance covered (m)	14.90 ± 2.96	15.28 ± 2.68	17.17 ± 2.48*

Mean ± SD. * = *P* > 0.05

There were significant improvements in anaerobic fitness over the training period (Table 2) with a 41.74 % increase in anaerobic power output and 42.11 % increase in relative power output over the training period *(p* < 0.05*)*. Peak speed increased 15.79 % and distance covered in 10 s increased 12.37 %.

There were no significant changes at any stage in static or dynamic postural control of the individuals *(p* < 0.05*)*. This was evident in the lack of significant change in sway path length, velocity and 100 % square of the individuals as seen in Table 3.

**Table 3:** Static and dynamic postural control results

Parameter	Initial test	Pre-training	Post-training
*Static Postural Control*			
EO SPL (mm)	2204.91 ± 549.52	2386.37 ± 674.90	2371.99 ± 477.80
EO Vel (mm/s)	44.10 ± 10.99	47.73 ± 13.50	47.44 ± 9.56
EO 100 % (mm^2^)	637.24 ± 346.77	1013.38 ± 948.25	572.47 ± 236.06
EC SPL (mm)	2474.62 ± 532.99	2601.18 ± 738.19	2600.05 ± 385.28
EC Vel (mm/s)	49.49 ± 10.66	52.02 ± 14.76	52.00 ± 7.70
EC 100 % (mm^2^)	1209.51 ± 460.81	1160.77 ± 467.17	1282.42 ± 781.40
*Dynamic Postural Control*			
EOAP SPL (mm)	1215.47 ± 238.58	1266.98 ± 344.81	1271.89 ± 180.44
EOAP Vel (mm/s)	48.62 ± 9.54	50.68 ± 13.79	50.88 ± 7.22
EOAP 100 % (mm^2^)	815.24 ± 307.75	986.78 ± 385.58	823.74 ± 268.32
ECAP SPL (mm)	1961.72 ± 463.10	2060.71 ± 623.97	1914.60 ± 468.95
ECAP Vel (mm/s)	78.47 ± 18.52	82.43 ± 24.96	76.58 ± 18.76
ECAP 100 % (mm^2^)	4360.57 ± 2660.85	4508.89 ± 2556.87	4774.32 ± 3515.18
EOML SPL (mm)	1325.47 ± 274.27	1374.11 ± 381.81	1352.98 ± 166.99
EOML Vel (mm/s)	53.02 ± 10.97	54.97 ± 15.27	54.12 ± 6.68
EOML 100 % (mm^2^)	952.56 ± 521.14	1175.98 ± 517.55	988.34 ± 687.01
ECML SPL (mm)	2282.17 ± 774.32	2271.02 ± 835.68	2137.33 ± 579.63
ECML Vel (mm/s)	91.29 ± 31.05	90.84 ± 33.43	85.49 ± 23.19
ECML 100 % (mm^2^)	5757.19 ± 4831.81	6071.60 ± 5639.39	4978.82 ± 2524.22

where *EO* eyes open, *EC* eyes closed, *AP* anterior posterior instability, *ML* medial lateral instability, *SPL* sway path length, *Vel* velocity of sway Results are expressed as mean ± SD

**Fig. 3:**
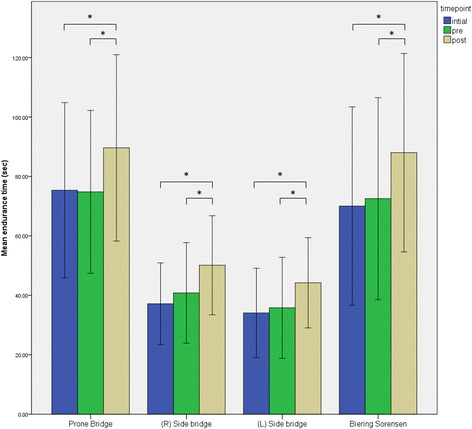
Results of the isometric trunk endurance tests. * = significant difference, *p* < 0.05

Figure 3 shows significant (*p* < 0.05) increases for all strength tests of the core muscles post intervention with a 19.78 % increase in the prone bridge and 22.84 % and 23.45 % increase in the left and right bridges respectively. The Biering Sorensen improved 21.33 % post intervention. There were no significant differences between the right and left sided bridges during any stage of testing.

**Fig. 4:**
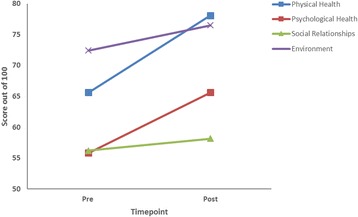
Results of the self-rated Quality of Life Questionnaire

Quality of life measures were significantly improved across a number of areas (Fig. 4). An increase of 9.95 % of the self-rated quality of life over the 6 weeks was seen along with a significant improvement of 28.05 % of self-satisfaction with the participants own health (*p* < 0.05). The first two domains of Physical Health (+18.99 %) and Psychological Health (+17.49 %) improved significantly (*p* < 0.05) while the other domains of Social Relationships and Environment exhibited improvements of +3.41 and +5.62 % respectively (Fig. 4).

## Discussion

This was the first study to assess the effects of participation in a training intervention of SUP. The aim of this study was to gain an insight into the effects of SUP on fitness, balance, strength and self-rated quality of life on the previously untrained individual.

The slight increase in HDL has been seen previously with aerobic exercise and normally increases in a dose dependent manner [17]. Previous increases of 4–22 % in HDL and decreases of 4–37 % of triglycerides have been reported with aerobic exercise including walking, jogging, swimming and cycling [18]. Correlations have been seen between training volume and HDL changes in walking jogging and running amongst healthy men [18] and previous studies have reported that 12 weeks is normally the timeframe to see significant changes in HDL, body composition and body weight, perhaps while no significant changes were seen here.

Plasma LDL is normally not lower after aerobic exercise [19] and is often elevated in people who have a high dietary fat intake [20]. Previous investigations suggest that dietary changes in conjunction with exercise do have the capacity to improve total cholesterol, LDL and triglycerides, while exercise alone primarily can have an effect of triglycerides only [21]. As the dietary habits were not changed in this intervention, the insignificant change in LDL was expected.

Participants in this study were advised to not change any dietary or training habits outside of the SUP training in order to minimize confounding factors on lipids and body composition. Greater body composition changes and HDL changes have previously been reported when combining caloric restriction and exercise training together [22]. This study was restricted to 6 weeks for funding and participant compliance reasons although it is assumed a longer training intervention amongst this group would further magnify the changes seen. A training intervention specifically targeted at people with a poor lipid profile would be necessary to effectively evaluate SUP as a training tool to change lipid profiles.

Previous studies have utilized various training interventions such as running and cycle ergometry on sedentary populations to ascertain benefits on fitness. The 23.57 % increase in relative VO_2_max this study are comparable to the 33 % increase found after high intensity walking/running training at 85–95 % HRmax, three times per week [23] and the 22.3 % increases in VO_2_max over a 12 week training intervention performed three times per week utilizing a cycle ergometer at low intensity of 60–80 % W_max_ [24].

Shorter studies over 6 weeks have found increases of 20.6 % utilizing cycle ergometry at 95%VO_2_ reserve, three times per week [25]. Other interventional studies have found increases as little as 7.4 % [26], 8.4 % [27] and 2.1 % [28]. Overall, intermittent, higher intensity training has been shown to elicit greater increases in aerobic power than continuous training over a longer duration.

The large increase (%) found in VO_2_max may be explained by the volume of training (180mins/week) and the incorporation of both long-slow low intensity and interval high intensity intermittent training throughout the training program. The incorporation of the higher intensity training would explain the large increases in anaerobic fitness from this study. The increases in anaerobic power are indicative of an increased capacity of the short term energy systems (ATP-PC). As there was a progressive increase in intensity of training as the program progressed, this would provide an ongoing stimulus for adaptation.

Another explanation for the significant improvement seen in maximal aerobic capacity may be the low baseline fitness levels observed prior to initiation of the training intervention. Other studies have shown significant increases in VO_2_max when low baselines were recorded [24]. Additional evidence of adaptation of the cardiovascular system from this study include the significantly greater distance covered over the duration of the post intervention test, a significantly greater peak aerobic speed and significantly greater peak aerobic power output.

The increases in trunk muscle endurance found in this study are similar to a previously published study utilizing the Swiss ball, where increases in endurance hold times of 30.34 % of the extensors of the spine and 57.05 % in the side bride have been found after 10 weeks of training [29]. Another study found an increase of 45.95 % in trunk endurance with a 12 week Swiss ball training program [30]. It is assumed that a longer training period on SUP would elicit similar gains.

Surprisingly there was no significant change in the static and dynamic balance capabilities of the participants in this study. Previous papers have shown the benefit of balance training on ankle instability subjects over a 6 week duration indicated by a decreased sway path length measured on a force plate [31]. A study on healthy individuals did show a decrease in both AP & ML parameters of sway over 10 weeks of balance training but was tested in single leg stance [32]. For the purposes of specificity, double leg stance was chosen for the balance assessment of the SUP participants. This result could be due to the training period not being long enough to elicit measurable balance benefits or as has been suggested previously, unstable surface training may not be effective for those people who do not have a balance deficit [33]. It could also be that the testing protocol is not specific to the demands of this sport and therefore unable to identify any adaptation which may have occurred over the 6-week training program. Although our previous research has shown superior balance ability from an elite population of SUP athletes [8], it may be that individuals that already possess a high level of balance are attracted to and subsequently succeed in such sports.

Significant improvements in self-rated quality of life measures are in agreement with studies which have found associations between physical activity and quality of life [34]. Although this was not a group recruited for their quality of life specifically, associations between inactivity and poor self-rated quality of life have been reported previously [34]. As most of the previous studies utilizing self-rated quality of life measures have been made with populations with health problems, direct comparison of initial and final results is difficult. One study did stratify age and sex with normal values amongst Danish citizens [35]. To use the values from this study which were 77 in Domain 1, 69 in Domain 2, 69 in Domain 3 and 74 in Domain 4, the results at the end of the study compare well to these figures. The primary change in quality of life in this study was in the physical and psychological domains indicative of positive changes in activities of daily living, dependence on substances and aids, energy and fatigue, mobility, pain and discomfort, sleep and rest and work capacity. Positive psychological aspects in the second domain include body image and appearance, negative and positive feelings, self-esteem, personal beliefs and thinking, learning, memory and concentration. As there was no direct influence on environmental or social aspects, the lack of significant change in these areas was expected.

Although it wasn’t the primary aim of this study, one participant did report a 20 mmHg drop in systolic blood pressure (SBP) over the duration of the training intervention. Previous studies have demonstrated the effect of both resistance and aerobic training on blood pressure with reductions in SBP from 141 to 136 mmHg with aerobic training (30mins treadmill at 65 % VO_2_max 3×/week for 4 weeks) and 136 to 132 mmHg with resistance training (machine circuit at 65 % 1RM 3×/week for 4 weeks) [36]. This is clearly in need of more research with higher numbers, but given the other associated health benefits, it is not unreasonable to have a positive effect of blood pressure utilizing this sort of activity.

There are some limitations to this study which need to be acknowledged. The study had a relatively small sample size and small duration due to limitations in funding and time. Although there was no independent control group, the participants acted as their own controls in the initial 6 weeks of this study. Future studies could include a longer intervention, with greater participant numbers and an independent control group. It is suggested that another measure of the postural control of SUP be utilized as the non-significant change is this study is surprising and possibly due to a lack of sensitivity in which balance was assessed.

The multitude of benefits from participation in SUP should be acknowledged. Currently sedentary behaviors contribute to ischemic heart disease, stroke, type 2 diabetes, kidney disease, arthritis, osteoporosis, colorectal cancer and depression [37] and a common barrier to exercise is a perceived lack of time and a dislike of exercise [5]. The fact that many physiological, musculoskeletal and psychological benefits can be obtained from participation places SUP as an ideal option for those who are time limited and still looking to improve strength and fitness. Due to it being accessible, relatively easy to learn and low impact on the joints is also of great benefit. The obvious psychological benefits and enjoyment obtained from this activity delivers an alternate means of aerobic, anaerobic and strength training than the traditional methods.

## Conclusion

Stand up paddle boarding appears to be an enjoyable, easy to learn alternative to traditional forms of training. This study shows significant improvement in aerobic and anaerobic fitness, multidirectional trunk endurance and self-rated quality of life measures can be elicited by SUP participation for previously untrained individuals. This study provides some evidence for the anecdotal claims of benefits of its participation.

## Abbreviations

AP: Anterior posterior
COP: Centre of pressure
EC: Eyes closed
EO: Eyes open
HDL: High density lipoproteins
LDL: Low density lipoproteins
ML: Medial lateral
SBP: Systolic blood pressure
SPL: Sway path length
SUP: Stand up paddle boarding
TC: Total cholesterol
Trigs: Triglycerides
VO2 max: Maximal aerobic power
WHO-QoL: World Health Organisation quality of life
